# Pathways shaping the mitochondrial inner membrane

**DOI:** 10.1098/rsob.210238

**Published:** 2021-12-01

**Authors:** Till Klecker, Benedikt Westermann

**Affiliations:** Institut für Zellbiologie, Universität Bayreuth, 95440 Bayreuth, Germany

**Keywords:** ATP synthase, cristae, Mgm1, MICOS, mitochondrial lipids, *Saccharomyces cerevisiae*

## Abstract

Mitochondria are complex organelles with two membranes. Their architecture is determined by characteristic folds of the inner membrane, termed cristae. Recent studies in yeast and other organisms led to the identification of four major pathways that cooperate to shape cristae membranes. These include dimer formation of the mitochondrial ATP synthase, assembly of the mitochondrial contact site and cristae organizing system (MICOS), inner membrane remodelling by a dynamin-related GTPase (Mgm1/OPA1), and modulation of the mitochondrial lipid composition. In this review, we describe the function of the evolutionarily conserved machineries involved in mitochondrial cristae biogenesis with a focus on yeast and present current models to explain how their coordinated activities establish mitochondrial membrane architecture.

## Graphical abstract

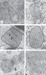

## 1. Introduction

Mitochondria are known as the ‘cellular powerhouses’ that produce ATP by oxidative phosphorylation. They are the site of numerous metabolic pathways, including β-oxidation of fatty acids, biosynthesis of haem, certain phospholipids and other metabolites. Furthermore, they are strictly required for the assembly of iron–sulfur clusters, which are essential cofactors of mitochondrial and non-mitochondrial enzymes. They are key regulators of programmed cell death (apoptosis) and participate in developmental processes and cellular ageing [1]. This variety of different functions of mitochondria is reflected in their complex architecture [2,3]. Their double membrane-bounded nature was revealed by the advent of cellular electron microscopy in the 1950s [4]. The mitochondrial outer and inner membranes enclose two aqueous compartments: the intermembrane space and the matrix. The inner membrane is subdivided into the inner boundary membrane, which runs parallel to the outer membrane, and cristae membranes. Cristae are characteristic folds of the inner membrane that penetrate into the matrix. High-resolution three-dimensional imaging of mitochondria by electron tomography revealed in the 1990s that cristae are often connected to boundary regions by relatively uniform narrow openings, termed crista junctions [5,6]. Proteinaceous contact sites physically connect the inner with the outer membrane [7,8].

The inner boundary membrane and cristae are functionally differentiated and have a distinct protein composition. Respiratory chain complexes and proteins involved in the assembly of iron–sulfur clusters are enriched in cristae membranes, whereas the protein translocation and membrane fusion machineries are mainly present in the inner boundary membrane [9–12]. Strikingly, the assembly of respiratory chain complexes is spatially orchestrated at distinct sites of the inner membrane; early steps of complex III and IV assembly occur in the inner boundary membrane, whereas the ATP synthase (complex V) is assembled and functions exclusively in cristae membranes [13].

The compartmentalization of respiratory chain complexes in cristae membranes is thought to enhance respiratory efficiency. The respiratory chain pumps protons from the matrix into the intermembrane space to generate a membrane potential, ΔΨ, which then fuels the ATP synthase. Confinement of these processes to cristae has been suggested to create a proton sink in the intra-cristal space which optimizes the performance of the ATP synthase [14]. Furthermore, respiratory chain complexes are organized in supercomplexes that allow efficient electron flux [15–17]. Cristae serve to enhance assembly and stability of the respiratory chain complexes and position them in close proximity [18].

**Figure 1.**
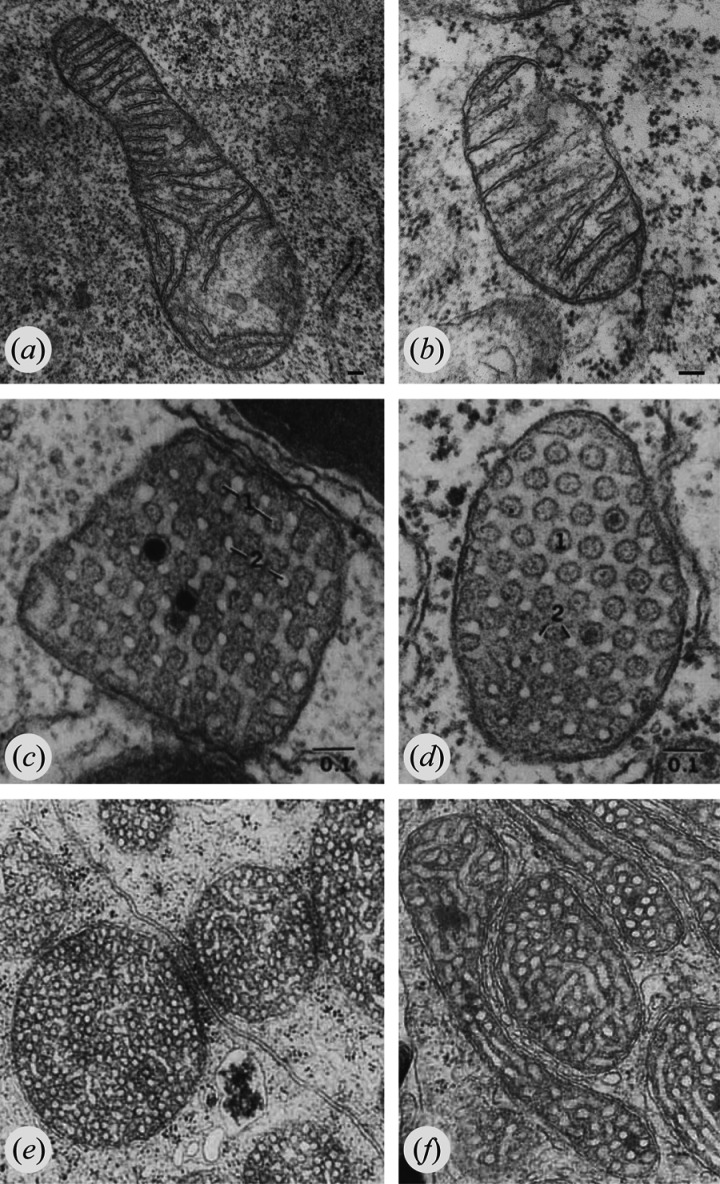
Mitochondrial cristae have varied morphologies. (*a*,*b*) Mouse embryonic fibroblast mitochondria contain the characteristic lamellar cristae that can be found in many cell types. Bars, 0.1 µm. Images courtesy of Beatrix Löwer and Stefan Geimer, Universität Bayreuth. (*c*,*d*) Mitochondria of sustentacular cells surrounding spermatozoa in *Xenopus laevis* contain cristae forming pleated folds pierced by regular rows of fenestrations. 1, fenestrations; 2, cross sections of tubular cristae elements. Bars, 0.1 µm. Images reproduced from reference [19] with permission from John Wiley and Sons. (*e*,*f*) Mitochondria of cells in the zona reticularis in the adrenal cortex of rats contain mostly tubulovesicular cristae. Images reproduced from [20] with permission from John Wiley and Sons. Copyright for (*c–f*) is held by John Wiley and Sons.

The morphology of mitochondrial cristae is highly varied and cell type-specific. Cristae frequently appear as lamellar structures that protrude more or less perpendicularly from the inner boundary membrane into the matrix space (figure 1*a*,*b*). Alternatively, they might form highly regular, fenestrated sheets (figure 1*c*,*d*) or abundant tubulovesicular structures (figure 1*e*,*f*). In some cells, cristae are short, tubular invaginations of the inner membrane and in other cells they form highly regular triangular or cubic structures. Some examples of extraordinary shapes of mitochondrial cristae can be found (e.g. [19–24]).

Cristae are highly dynamic structures. In the 1960s it was shown that cristae morphology of isolated rat liver mitochondria is extensively and reversibly remodelled with the energetic state. An excess of ADP induces a ‘condensed’ conformation with large, swollen intra-cristal space, whereas under ADP-limiting conditions mitochondria adopt the ‘orthodox’ conformation with contracted intra-cristal space, as it is usually observed in fixed tissues [25]. Mitochondria of the giant amoeba, *Chaos carolinensis*, normally contain randomly oriented tubular cristae. Upon starvation, cristae become enlarged and adopt a cubic morphology and a zigzag-like pattern [24,26]. Exposure of murine mitochondria to the pro-apoptotic protein tBID induces another striking ultrastructural change. Individual cristae fuse and crista junctions open to release cytochrome *c* from the intra-cristal space into the boundary region. From there it can be efficiently discharged to the cytosol upon outer membrane permeabilization to trigger the cell death machinery [27].

Recent advances in live cell super-resolution light microscopy enabled the analysis of the dynamic behaviour of cristae in mammalian mitochondria (reviewed in [28]). It was found that individual cristae are functionally independent and display different membrane potentials, even within the same mitochondrion [29]. Furthermore, cristae membranes undergo continuous cycles of remodelling [30] and cristae of two mitochondria may merge into one object upon mitochondrial fusion [31]. The biogenesis of cristae presumably involves both remodelling of pre-existing unstructured cristae precursors and de novo formation of crista junctions on existing cristae [32].

In the past decade, considerable progress has been made in elucidating and mechanistically analysing the molecular machinery that shapes the mitochondrial inner membrane. The biogenesis of mitochondrial cristae largely depends on the coordinated activities of four major pathways: dimer formation and oligomerization of the ATP synthase at cristae rims, assembly of the ‘mitochondrial contact site and cristae organizing system’ (MICOS) at crista junctions, membrane remodelling by an inner membrane-associated dynamin-related GTPase (Mgm1 in yeast and OPA1 in mammals) and proper adjustment of the membrane lipid composition (tables 1 and 2). As these pathways have been conserved during evolution, they can be studied in a wide range of organisms from protists and yeasts to higher eukaryotes and humans. In particular baker's yeast, *Saccharomyces cerevisiae*, has enabled many pioneering discoveries in the identification and functional characterization of the conserved cristae-shaping machineries and has remained a major model organism to this day. Hence, we shall focus this review on the conserved pathways of cristae biogenesis in yeast, of course with mentioning important discoveries made in other organisms. Recent reviews have elaborated on the mammalian orthologues and their roles in human disease (e.g. [81–87]).

**Table 1.** Core machinery involved in cristae formation. Yeast standard names are according to *Saccharomyces* Genome Database [33]; the nomenclature of MICOS subunits is according to reference [34]; alternative protein names are in brackets.

yeast	human orthologue	proposed function	selected references
*ATP synthase*			
Atp4 (Su b; subunit 4; Ypl078c)	ATP5F1 (subunit b)	subunit of the stator stalk; ATP synthase dimerization	[35–37]
Atp20 (Su g; Ypr020w)	ATP5L (subunit g)	ATP synthase dimerization	[36–41]
Atp21^a^ (Tim11^a^; Su e; Ydr322c-a)	ATP5I (subunit e)	ATP synthase dimerization	[35,37–42]
*inner membrane-associated dynamin-related GTPase*			
Mgm1 (Yor211c)	OPA1	inner membrane fusion, generation of lamellar cristae	[42–45]
*MICOS subunits*			
Mic10 (Mcs10; Mio10; Mos1; Ycl057c-a)	MIC10 (MINOS1)	membrane-shaping core subunit of Mic10 subcomplex	[46–51]
Mic12 (Aim5; Fmp51; Mcs12; Ybr262c)	MIC12 (MIC13; QIL1)	coupling of Mic10 and Mic60 subcomplexes	[46–48,52]
Mic19 (Aim13; Mcs19; Yfr011c)	MIC19 (CHCHD3; MINOS3) and MIC25 (CHCHD6)	assembly and regulation of Mic60 subcomplex	[46–48,53–55]
Mic26 (Mcs29; Mio27; Mos2; Ygr235c)	MIC26 (APOO; MIC23)	destabilization of Mic10 oligomers	[46–48,56]
Mic27 (Aim37; Mcs27; Ynl100w)	MIC27 (APOOL)	stabilization of Mic10 oligomers	[46–48,52]
Mic60 (Aim28; Fcj1; Fmp13; Ykr016w)	MIC60 (HMP; IMMT; MINOS2; mitofilin)	membrane-shaping core subunit of Mic60 subcomplex	[38,46–48,53,54,57–60]

^a^The standard gene name is *TIM11* (translocase of the inner mitochondrial membrane of 11 kDa) according to *Saccharomyces* Genome Database [33]. Tim11 was initially thought to be involved in the sorting of precursor proteins to the mitochondrial intermembrane space [61]. However, it turned out that this protein in fact is identical to subunit e of the ATP synthase [62]. As a role in ATP synthase dimer formation is well established we prefer in this review the gene name *ATP21* (ATP synthase 21) [39].

**Table 2.** Mitochondrial phospholipid homeostasis and cristae structure in yeast. Major alterations of the mitochondrial phospholipid profile and cristae structure that were observed in mutants are listed. OE, analysed upon overexpression. Alternative protein names are in brackets.

protein	mitochondrial lipid profile in mutant	cristae structure of mutant	selected references
*lipid transport*			
Mdm31 (Yhr194w)	PE and CL reduced	altered	[63,64]
Mdm32 (Yor147w)	CL reduced	altered	[63,64]
Mdm35 (Ykl053c-a)	PE reduced	wild-type-like, less cristae	[45,63,65,66]
Mmm1 (Yme6; Yll006w)	PE and CL reduced	altered	[63,67,68]
Ups1 (Ylr193c)	CL reduced	wild-type-like	[63,69,70]
Ups2 (Aim30; Gep1; Msf1; Ylr168c)	PE reduced	wild-type-like, less cristae	[63,65,66,69,71]
*CL biosynthesis and remodelling*			
Cld1 (Ygr110w)	altered CL acyl chain composition	wild-type-like, elongated cristae	[72,73]
Crd1 (Cls1; Ydl142c)	CL reduced	wild-type-like, elongated cristae	[63,72,74]
Gep4 (Yhr100c)	CL reduced	n.d.	[63]
Pgs1 (Pel1; Ycl004w)	CL reduced, PA and CDP-DAG increased	altered	[70]
Tam41 (Mmp37; Ygr046w)	CL reduced, PA increased	wild-type-like	[70]
Taz1 (Ypr140w)	MLCL increased, CL decreased	wild-type-like, elongated cristae	[72,74]
*PE biosynthesis*			
Psd1 (Ynl169c)	PE reduced	wild-type-like, reduced cristae length	[63,75–77]
*PC biosynthesis*			
Opi3 (Pem2; Yjr073c)	PC reduced, PE increased	wild-type-like	[75]
Cho2 (Pem1; Ygr157w); Opi3 (Pem2; Yjr073c)	PC reduced in Δ*cho2 Δopi3* double mutant	wild-type-like in Δ*cho2* Δ*opi3* double mutant	[78]
Cki1 (Ylr133w); Dpl1 (Bst1; Ydr294c); Eki1 (Ydr147w)	PC and PE reduced in Δ*cki1* Δ*dpl1* Δ*eki1* triple mutant	wild-type-like in Δ*cki1* Δ*dpl1* Δ*eki1* triple mutant	[78]
*other*			
Mdm33 (She9; Ydr393w)	PE and CL reduced (OE)	altered (OE)	[79,80]

## 2. Mitochondrial architecture in yeast

*Saccharomyces cerevisiae* is a powerful model organism to study mitochondrial biology because it can satisfy its energy requirements either by glycolytic fermentation or by mitochondrial respiration. Fermentation of glucose is the preferred metabolic pathway, and mitochondrial respiration is dispensable as long as fermentable carbon sources are available. Under these conditions, most genes required for oxidative phosphorylation are repressed. When fermentable carbon sources become limiting, the expression of genes required for respiration is induced and ATP is produced from the metabolism of non-fermentable substrates, such as glycerol, ethanol or lactate [88,89]. The yeast mitochondrial genome (mtDNA) encodes seven subunits of the respiratory chain and one subunit of the mitochondrial ribosome. Mutants lacking mtDNA, termed cytoplasmic *petite* or [*rho^0^*], are respiratory-deficient [90]. In addition, at least 254 nuclear genes are crucial for respiratory metabolism of yeast [91].

**Figure 2.**
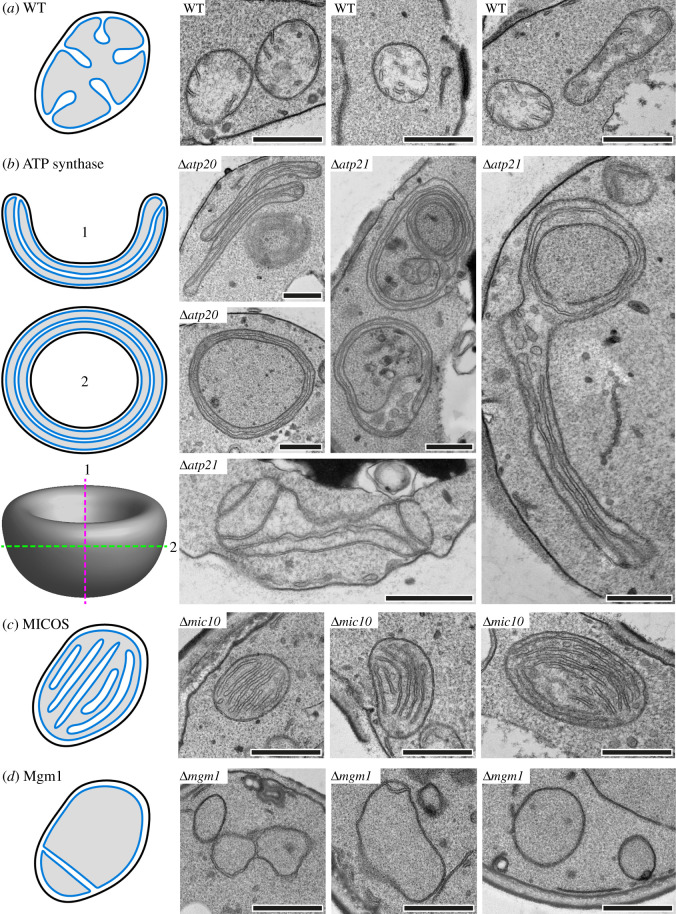
Mitochondrial ultrastructure is altered in yeast cells lacking components of the core machinery involved in cristae formation. The cartoons on the left side illustrate mitochondrial ultrastructure in (*a*) wild-type (WT) yeast cells or mutants lacking, (*b*) ATP synthase dimerization, (*c*) MICOS or (*d*) Mgm1. On the right side, representative images of mitochondria from yeast cells of the indicated genotypes are shown. Chemical fixation and sample preparation for transmission electron microscopy was performed as described in reference [95]. Scale bars: 500 nm. In (*b*) a model is shown to illustrate how phenotypes of ATP synthase dimerization mutants (i.e. onion-like and septated mitochondria) could originate from the same cup-shaped organelle, depending on the way it is sectioned, as was previously suggested by Velours *et al*. [96].

Already in the 1960s, electron microscopic studies revealed the ultrastructure of mitochondria in *S. cerevisiae* [92]. The outer and inner boundary membranes are closely apposed. Cristae protrude from the inner membrane into the matrix at irregular intervals. They are mostly lamellar, tubular or bell-shaped and extend only partially across the mitochondrion. During fermentative growth on glucose-containing medium, mitochondria have relatively few, poorly defined cristae and many mitochondrial sections show no cristae at all. During respiratory growth on non-fermentable carbon sources, the amount of mitochondria increases and cristae become more prominent [92,93]. Some mitochondria were found to contain concentric lamellar cristae in stationary cells [92]. In the 1970s, three-dimensional models constructed from serial sections of entire yeast cells demonstrated that mitochondria form large networks consisting of branched tubules [94]. The average cross-sectional diameter of mitochondrial tubules is about 300–400 nm [93]. More recently, the analysis of ultrathin sections and electron tomograms of wild-type cells revealed crista junctions, the diameters of which typically range from 12 to 25 nm [38,46]. Focused ion beam milling combined with scanning electron microscopy (FIB-SEM) showed that cristae are often arranged in a helical pattern running along mitochondrial tubules [57]. Characteristic morphologies of cristae in wild-type yeast mitochondria are shown in figure 2*a*.

The electron microscopic studies suggest that respiratory growth induces the biogenesis of cristae to accommodate the respiratory chain complexes, whereas the number and size of cristae are reduced when respiratory chain complexes are lacking. This was supported by a recent study that quantified the changes of the mitochondrial proteome upon transition from fermentative to respiratory metabolism [97]. Intriguingly, the protein levels of ATP synthase, MICOS and Mgm1 are all strongly upregulated upon transition to respiratory growth. Their abundance is increased about two to threefold in relation to mitochondrial dry weight, indicating that the increase does not merely reflect the increase in mitochondrial mass. These observations suggest that enhanced biogenesis and remodelling of cristae membranes prepare the mitochondria for maximum respiratory function during growth on non-fermentable carbon sources [97].

Mitochondria of [*rho^0^*] mutants lack respiratory chain complexes. They are largely devoid of cristae, and some organelles contain septa that extend across the entire organelle and separate the matrix [93,98–101]. Similar ultrastructural phenotypes can also be found in mitochondria of respiratory-deficient mutants carrying nuclear gene deletions [102]. However, anaerobically grown cells contain at least some mitochondrial cristae [103], and some respiratory-deficient mutants were found to have normal mitochondrial ultrastructure [100,104]. This suggests that a lack of respiratory activity does not generally lead to ultrastructural phenotypes or the absence of cristae.

## 3. ATP synthase dimerization

The establishment of the fine structure of the mitochondrial inner membrane is based on elaborate sculpturing of the membrane. This results in regions with high membrane curvature, in particular at crista junctions and cristae tips. One main contributor to cristae structure is the F_1_F_o_-ATP synthase, which is best known for its role in metabolic energy generation. Several observations that led to the elucidation of the role of the ATP synthase in shaping cristae were initially made in yeast. However, important contributions also came from studies in mammals, the colourless algae *Polytomella* sp. and other organisms.

A first hint that the ATP synthase could be involved in establishing cristae structure came from observations made in the protist *Paramecium multimicronucleatum*. Rapid-freeze deep-etch electron microscopy revealed double-rowed arrays of ATP synthase complexes running along tubular cristae [105,106]. This intriguing discovery led to the idea that the organization of these complexes into higher-order structures might drive the formation of tubular cristae [105].

Indeed, the assembly of the ATP synthase into supercomplexes turned out to be very important for mitochondrial ultrastructure. It was first shown by blue native gel electrophoresis that the ATP synthase from *S. cerevisiae* forms dimers [39]. Higher-order oligomers were also detected in mitochondria solubilized with mild detergent [35,36,40]. Two-dimensional gel electrophoresis revealed that three subunits are specifically associated with the dimeric form, namely Atp19 (subunit k), Atp20 (subunit g) and Atp21 (subunit e, alternative name Tim11) [39]. Of these, Atp20 and Atp21 are strictly required for ATP synthase dimerization [39] and formation of ATP synthase oligomers [35,40,41]. Strikingly, Δ*atp20* and Δ*atp21* mutants contain mitochondria with strongly altered ultrastructure [35,40] (figure 2*b*). Similarly, cells lacking the first transmembrane domain of Atp4 (subunit b, also referred to as subunit 4) show loss of ATP synthase dimers and oligomers [35,36] as well as irregularly shaped cristae membranes [36]. Importantly, assembly of active monomeric F_1_F_o_-ATP synthase is still possible in all three mutants [36,39,40]. Together, these results indicate a strong dependence of cristae structure on ATP synthase supercomplex formation.

How does dimerization and oligomerization of the ATP synthase contribute to the formation of mitochondrial ultrastructure? The answer to this question probably lies in the shape adopted by the dimer. In several organisms, including yeast, the two monomers are not oriented in parallel but are instead tilted against each other and adopt a wedge or so-called V shape [107]. This was first observed by single-particle electron microscopy of isolated ATP synthase dimers from bovine heart [108], algae (*Polytomella*) [109] and yeast [110]. Different tilt angles ranging from approximately 35–140° were observed for isolated yeast ATP synthase dimers [110–112]. However, cryo-electron tomography of isolated mitochondrial membranes consistently detected dimers with a tilt angle of 80–86° [37,113], which closely matches the initially proposed angle of approximately 90° [110] and probably reflects the predominant form in yeast mitochondria. While subunit composition and shape of the dimeric ATP synthase are similar in yeast and mammals, it should be noted that they might diverge in other organisms (see [107] for more information).

The ATP synthase is deeply embedded in the membrane with its F_o_ part. Thus, it is conceivable that dimer formation causes massive deformation of the membrane due to the high tilt angle between the monomers. In agreement, a recent high-resolution cryo-electron microscopy structure of the F_o_ part of dimeric yeast ATP synthase indicates that the dimer bends the membrane almost by a right angle [114]. The structure revealed that Atp6 (subunit a) and Atp18 (subunit i/j) from both monomers form direct contacts at the monomer–monomer interface. Atp19 and Atp21 contribute to the dimerization and Atp4, Atp20 and Atp21 together are presumably responsible for the tilt angle between the monomers [114].

Higher-order oligomers of ATP synthase can be readily detected by native polyacrylamide gel electrophoresis [35,36,40]. The size distribution of oligomers isolated from rat heart mitochondria indicated that they are formed by association of dimers [115], which was later also confirmed for yeast [116]. Consistently, oligomers cannot be observed in yeast mutants lacking functional dimerization-promoting ATP synthase subunits, including Atp20 [35,36,40], Atp21 [35,40,117] or Atp4 [35,36]. Cross-linking experiments in yeast resulted in the formation of both homo- and heterodimers of Atp20 and Atp21 [117–119]. Strikingly, in both cases the cross-linked homodimers were associated with ATP synthase oligomers [117,118]. Atp4, Atp19, Atp20 and Atp21 are located laterally at both long sides of the dimer [114]. Thus, the observed cross-linked Atp20 and Atp21 homodimers are probably based on inter-dimer interactions, indicating that within an oligomer the dimers are oriented in parallel and stack side by side. In agreement, rows of ATP synthase dimers have been observed in yeast by freeze-fracture electron microscopy of isolated mitochondria [112], as well as atomic force microscopy [120], cryo-electron tomography [113] and negative stain electron microscopy [112] of isolated mitochondrial membranes. Within these rows the dimers were found to be arranged in parallel and stack laterally [112,113]. Taken together, rows of ATP synthase that are formed by dimers stacking side by side along their lateral interfaces (i.e. perpendicular to the monomer–monomer interface) appear to represent the oligomeric form in yeast. Rows of ATP synthase have also been observed in various other organisms, including protists, green algae, higher plants, fungi and animals [14,105,106,113,121,122].

ATP synthase dimer rows are not randomly distributed within the membrane, but associated with highly curved membrane regions. In several organisms, they were found to decorate the tapered rim of mitochondrial membrane vesicles [14,37,112,113] and line tightly bent edges of cristae membranes in isolated mitochondria [113,122]. In agreement, yeast Atp20 and Atp21 were found to be preferentially localized to the cristae tips by immunogold electron microscopy [38]. ATP synthase dimers distort the membrane due to the high tilt angle between the monomers. Thus, rows of dimers that are arranged in parallel will kink the membrane along the longitudinal axis of the row. Accordingly, it has been suggested that rows of ATP synthase dimers generate high membrane curvature in cristae membranes [14]. However, it is not completely understood whether the association of rows of ATP synthase dimers with strongly bent membrane regions is the cause or consequence of the high curvature.

Molecular dynamics simulations suggest that the formation of linear arrays of dimers could be a self-driven process. Based on these simulations, ATP synthase dimers distort the membrane and the formation of rows of dimers is energetically favourable as it relieves membrane tension along the long axis of the row [37,123]. In agreement with this model, in yeast row formation and the preferential association of the ATP synthase with the rim of mitochondrial membrane vesicles is lost in the absence of Atp20 (i.e. the dimerization subunit g [37]). Furthermore, reconstitution of ATP synthase dimers from *Polytomella* sp. or the yeast *Yarrowia lipolytica* into liposomes results in the formation of linear dimer arrays associated with strong membrane deformation. Strikingly, similar alterations of liposome structure and row formation could not be observed when isolated monomeric *Y. lipolytica* ATP synthase was reconstituted into the membrane [122]. In sum, these observations indicate that rows of ATP synthase dimers are sufficient to induce membrane curvature, and dimerization is strictly required for both row formation and membrane deformation.

Yeast cells lacking Atp20, Atp21 or the first transmembrane domain of Atp4 exhibit loss of ATP synthase dimers [35,36,39] and harbour dramatically altered mitochondria that adopt onion-like shapes and contain elongated cristae that run along their longitudinal axis [35,36,40] (figure 2*b*). Furthermore, the elongated cristae membranes of Δ*atp20* and Δ*atp21* mutants were found to frequently be devoid of rounded tips. Instead, mutant cristae are sometimes branched and often connected to more than one crista junction [38]. Downregulation of *ATP21* expression by use of a doxycycline-controllable promoter revealed that initially parallel running elongated sheets of inner membrane develop before onion-like mitochondria appear [41], indicating a gradual decay of mitochondrial ultrastructure. Of note, the onion-like profiles seen in two-dimensional sections could originate from cross sections through cup-shaped mitochondria containing spread-out sheets of unfolded inner membrane [96] (figure 2*b*). Cryo-electron tomography of isolated mitochondria from dimerization-defective mutants furthermore revealed that the inner membrane is partially dissipated into adjoining vesicles [37]. This could mean that the elongated cristae sheets found in thin sections correspond to apposed limiting membranes of adjacent inner membrane vesicles.

According to the current model for the role of the ATP synthase in mitochondrial architecture, dimer rows associate with the highly curved cristae edges where they stabilize and/or cause high membrane curvature. As a consequence, highly curved regions such as cristae tips become unstable in the absence of dimeric ATP synthase. It has been hypothesized that lack of ATP synthase dimerization could lead to cristae fusion [96]. This in turn could explain the formation of branched and elongated inner membrane sheets and inner membrane vesiculation in ATP synthase dimerization-defective mutants.

## 4. Mitochondrial contact site and cristae organizing system

Mic60 (see table 1 for alternative names) was the first MICOS component with a known role in mitochondrial architecture. The human gene was initially cloned because of its high expression in the heart [124]. The first molecular characterization showed that human MIC60 is a ubiquitously expressed mitochondrial protein mainly located in the periphery of the organelle [125]. The analysis of knock-down cells revealed striking defects of mitochondrial architecture: the inner membrane failed to form normal cristae and showed closely packed stacks of membrane sheets [126]. An analysis of the yeast mutant lacking Mic60 showed that mitochondria similarly exhibit concentric stacks of inner membrane sheets, and overexpression leads to increased formation of crista junctions and branching of cristae [38]. These observations firmly established a crucial role of Mic60 in cristae formation. If not mentioned otherwise, the following discussion of MICOS will refer to studies made in yeast.

In 2011, three groups reported independently of each other the identification of the MICOS complex. Harner *et al*. used an artificial fusion protein spanning both mitochondrial membranes as a marker for contact sites connecting the outer with the inner membrane. After stable isotope labelling by amino acids in cell culture (SILAC) they subjected mitochondrial membrane fractions to quantitative mass spectrometry and identified proteins that specifically co-fractionated with the marker in density gradients. The contact site fractions contained Mic60 together with five new proteins that showed very similar gradient profiles [47]. Von der Malsburg *et al*. used a protein A-tagged Mic60 variant to identify interaction partners by affinity purification and mass spectrometry of SILAC-labelled mitochondrial extracts. They found two major components of the protein translocase of the outer membrane (TOM), Tom40 and Tom22, together with five novel proteins [48]. Hoppins *et al*. systematically quantified genetic interaction profiles of yeast genes associated with mitochondrial functions and found three novel genes clustering with *MIC60*. Pull-down experiments using epitope-tagged proteins yielded two additional interaction partners [46]. Astonishingly, all three of these fundamentally different experimental approaches revealed an identical set of Mic60 interaction partners: Mic10, Mic12, Mic19, Mic26 and Mic27 [46–48].

Mutants lacking either one of these proteins show striking defects of mitochondrial ultrastructure. The surface of the inner membrane is increased, stacks of lamellar cristae are formed, some cristae are extremely elongated and the number of crista junctions is strongly reduced [38,46–49] (figure 2*c*). The ultrastructural phenotype is particularly strong in Δ*mic10* and Δ*mic60*, and less pronounced in Δ*mic12*, Δ*mic19*, Δ*mic26* and Δ*mic27* [46–48]. All deletion mutants lacking MICOS subunits are able to grow on non-fermentable carbon sources [46,91], albeit some growth defects were reported for Δ*mic10* and Δ*mic60* [47–50]. Intriguingly, a yeast strain lacking all six MICOS subunits shows an exacerbated growth defect on ethanol/glycerol medium, suggesting that MICOS subunits have non-redundant functions. Consistently, defects of cytochrome *c* oxidase (respiratory chain complex IV) activity were apparent only when all MICOS subunits were absent, but not in a Δ*mic60* single mutant [53]. These observations demonstrate that MICOS plays a key role in establishing mitochondrial architecture. Mic10 and Mic60 are crucial, whereas the other components apparently play accessory roles. The ability of deletion mutants to grow on non-fermentable carbon sources indicates that normal cristae morphology is not absolutely required for the assembly and function of the respiratory chain.

Quantitative immunogold electron microscopy revealed that Mic60 is enriched at crista junctions [38]. Consistently, light microscopy of Mic19, Mic27 and Mic60 GFP fusions showed localization in discrete, punctate structures along mitochondrial tubules, while Mic12 and Mic26 were present mainly in extended filamentous structures that wrap around the inner membrane [46]. A recent study combined super-resolution light microscopy with FIB-SEM to analyse the spatial distribution of Mic60 in yeast and mammalian mitochondria with unprecedented resolution. MICOS was found to be arranged in two twisted bands of clusters that run along mitochondrial tubules, mirroring the helical arrangement of crista junctions [57]. Together, these observations suggest that MICOS is part of a complex structure establishing the internal architecture of mitochondria.

The MICOS core components, Mic60 and Mic10, form two separable subcomplexes [127,128]. The first subcomplex consists of Mic60 and Mic19. Mic60 is targeted to mitochondria by an N-terminal cleavable presequence, which is followed by a transmembrane segment anchoring the protein to the inner membrane. Its major part is exposed to the intermembrane space and contains a coiled-coil region, a lipid-binding site and a conserved C-terminal domain (also termed 'mitofilin domain') [38,54,58,59]. Mic60 is able to self-assemble into oligomeric structures in the absence of other MICOS subunits [53]. Mic19 is a peripheral membrane protein that interacts with the Mic60 'mitofilin domain', regulates Mic60 distribution within mitochondria and augments its activity [53,54]. Mic19 contains a cysteine-containing motif, which is oxidized *in vivo* and forms an intramolecular disulfide bond, raising the possibility that it functions as a redox-dependent regulator of MICOS [55].

The second MICOS subcomplex consists of the integral inner membrane proteins Mic10, Mic12, Mic26 and Mic27. Mic10 is a small, oligomeric protein with two transmembrane segments. It adopts a hairpin-like topology with both termini facing the intermembrane space and a short positively charged loop in the matrix [49–51]. Mic27 supports the stability of the MICOS complex [52,129]. However, its precise role is not entirely clear. On the one hand, it has been reported that Mic27 promotes the stability of Mic10 oligomers [52], and on the other hand, it was found that formation of a high molecular weight Mic10 complex does not require Mic27 [129]. Mic26 appears to have an antagonistic, destabilizing role [56]. Mic12 is a small transmembrane protein that promotes the coupling of the Mic10 subcomplex with Mic60 [52]. Assembly of Mic10 into the MICOS complex is also supported by Aim24, which is not an integral MICOS subunit itself [130].

Both Mic10 and Mic60 have membrane bending activities. Overexpressed Mic10 is able to form oligomers in mitochondria without the participation of other MICOS components. This induces an increase in the surface area of the inner membrane with highly interconnected and irregular formed cristae. These are connected to the inner boundary membrane by multiple junctions with aberrant shapes [51]. Reconstitution of purified Mic10 in artificial membranes results in the formation of tubular membrane structures with diameters between 10 and 30 nm in large unilamellar vesicles (LUVs), and internal vesicles and tubules in giant unilamellar vesicles (GUVs). This activity is dependent on oligomer formation of Mic10 [50]. Reconstitution of purified Mic60 also induces the formation of tubular membranes with diameters between 10 and 20 nm in LUVs, and internal vesicles and onion-like interconnected membrane layers in GUVs. Strikingly, expression of Mic60 in *Escherichia coli* induces invaginations of the plasma membrane, resembling mitochondrial cristae, and formation of intracellular vesicles. This activity resides in the intermembrane space domain, as Mic60 variants lacking the N-terminal transmembrane segment retain at least partial membrane remodelling activities in LUVs and *E. coli* [60]. A structure–function analysis of Mic60 of the fungus *Chaetomium thermophilum* suggests that the lipid-binding site is responsible for membrane bending *in vitro*, and this activity is negatively regulated by the 'mitofilin domain' [54]. In sum, these observations demonstrate that Mic10 oligomer formation and Mic60 membrane-binding activity are both sufficient to bend membranes *in vivo* and *in vitro*.

Mic60 physically interacts with two protein import and sorting machineries in the outer membrane: TOM, the major import pore for mitochondrial proteins synthesized by cytosolic ribosomes, and SAM (sorting and assembly machinery), which is required for insertion of β-barrel proteins into the outer membrane [47,48,59,131]. Furthermore, Mic60 transiently interacts with Mia40, an inner membrane protein promoting import and assembly of intermembrane space proteins by disulfide bond formation [48,132]. Moreover, an interaction of MICOS with the TIM23 preprotein translocase of the inner membrane was described [133]. It appears that preproteins imported via the Mia40 pathway and β-barrel proteins are most affected by the absence of Mic60. The molecular basis for the protein import defects observed in MICOS mutants is only poorly understood. It is possible that MICOS plays a direct, dual role in protein sorting. For example, Mic60 could promote the formation of membrane contact sites by binding to TOM and SAM and thereby support import via the Mia40 pathway. Also, Mic60 might stimulate the transfer of β-barrel precursors from TOM to SAM. However, it is also possible that protein import defects are indirect consequences of disturbed mitochondrial architecture [86,134,135].

As MICOS also interacts with other outer membrane proteins and outer membrane complexes are connected via inter-organelle contact sites to the ER it was proposed that MICOS is a central part of an ER–mitochondria organizing network, termed ERMIONE [136]. Consistently, it was found that both MICOS subcomplexes preferentially assemble at mitochondrial ER contact sites [137]. Assembly of the Mic10-containing subcomplex requires the function of ERMES, the ER–mitochondria encounter structure [138], whereas the Mic60-containing subcomplex is localized in proximity to the ER by an ERMES-independent mechanism [137].

MICOS has been highly conserved during evolution [139,140]. Human MICOS contains orthologues of all yeast subunits, and one additional component, MIC25 [34,82]. MIC25 is a paralogue of Mic19 in metazoan mitochondria [141,142]. Mic60 has been characterized in land plant mitochondria [143]. The euglenozoan *Trypanosoma brucei* contains two orthologues of Mic10, one Mic60-related protein and six additional subunits [144]. *MIC10-* and *MIC60*-related genes were found in the genomes of cristae-containing species in all eukaryotic kingdoms. Intriguingly, species lacking mitochondrial cristae are also lacking MICOS-related genes [145,146]. *MIC60*-related genes were even found in α-proteobacteria, the endosymbiotic ancestors of mitochondria [145,146]. Consistently, several α-proteobacteria show differentiated intracytoplasmic membrane structures [147,148]. Strikingly, expression of mitochondria-targeted Mic60 homologues of *Paracoccus denitrificans* and *Rhodobacter sphaeroides* partially rescues ultrastructural mitochondrial defects of Δ*mic60* yeast mutants [60]. Thus, during evolution of mitochondria Mic60 was derived from their endosymbiotic ancestors. Together with Mic10 it is present in most, if not all, cristae-containing mitochondria throughout all eukaryotic kingdoms, while homologues of Mic12, Mic19, Mic26 and Mic27 are consistently found among ophistokonts (i.e. the group containing animals and fungi) [145,146].

## 5. Inner membrane-associated dynamin-related GTPases

Mitochondria are highly dynamic organelles that continuously fuse and divide and adapt the shape of the mitochondrial compartment to the metabolic needs and physiological conditions of the cell. This requires coordinated fusion and fission of the mitochondrial outer and inner membranes. Details regarding the mechanisms and physiological roles of mitochondrial fusion and fission have been covered by several recent reviews (e.g. [149–154]). In brief, both mitochondrial fusion and fission are mediated by large dynamin-related GTPases. In yeast, the fission GTPase Dnm1 is recruited to mitochondria by the outer membrane protein Fis1 with the help of the soluble adapter proteins Mdv1 and Caf4. Dnm1 assembles into ring-shaped oligomers that enclose the mitochondria and constrict upon GTP hydrolysis, thereby severing the mitochondrial membranes. It is currently unknown whether a separate division machinery for the inner membrane exists. Mitochondrial fusion is mediated by two separate GTPases, Fzo1 in the outer and Mgm1 in the inner membrane. Fzo1 and Mgm1 are physically linked by the outer membrane protein Ugo1 to coordinate fusion of the outer with the inner membranes.

The *MGM1* gene (*m*itochondrial *g*enome *m*aintenance) was first identified in a screen for genes required for mtDNA maintenance [155]. Mutants lacking functional Mgm1 suffer from loss of mtDNA [155,156] and contain abnormal, fragmented and aggregated mitochondria [156–158]. Fluorescence microscopy-based mating assays revealed that Mgm1 is required for mitochondrial fusion in zygotes [43,157,159]. An analysis of mitochondrial fusion *in vitro* demonstrated that Mgm1 is specifically required for fusion of the inner membrane [44].

Several lines of evidence point to a specific role of Mgm1 and its metazoan homologue OPA1 in cristae formation, in addition to their role in inner membrane fusion. Fusion of the mitochondrial outer membrane without subsequent fusion of the inner membrane would result in mitochondria with separated, unfused matrix compartments. Indeed, such inner membrane septa are frequently observed by transmission electron microscopy in Δ*mgm1* mutants [42] and in mutants harbouring temperature-sensitive *mgm1* alleles [42,44]. This phenotype can likely be ascribed to the inner membrane fusion defect.

Strikingly, loss of functional Mgm1 is accompanied by additional, more severe defects of mitochondrial ultrastructure, resulting in a severe reduction of cristae number or the complete absence of cristae (figure 2*d*) [42–44,160]. Similarly, knockdown of *OPA1* in mammalian cells results in fragmentation of the mitochondrial network and abnormalities of mitochondrial ultrastructure. These include a complete lack of visible cristae or the appearance of irregularly shaped, enlarged or vesiculated cristae membranes, ring-like structures and widened crista junctions [161–163]. Mitochondria containing inner membrane septa and internal ring-like membranes have also been observed in *OPA1*-null mouse embryonic fibroblasts (MEFs) [164] and in worms carrying a mutant allele of *EAT-3* (the *Caenorhabditis elegans* homologue of *MGM1*) [165,166]. Thus, loss of *MGM1* or its homologues leads to a reduction in the number of cristae and the formation of inner membrane septa and ring-shaped inner membrane profiles in several organisms.

The understanding of Mgm1's contribution to the establishment of mitochondrial ultrastructure is complicated by its role in mtDNA maintenance. In yeast, three essential subunits of the membrane-embedded F_o_ part of the ATP synthase are encoded by mtDNA: Atp6, Atp8 and Atp9. Therefore, mutants lacking mtDNA are devoid of fully assembled ATP synthase and instead contain soluble F_1_ complexes [167–169]. Consequently, these mutants lack the membrane-shaping activity of ATP synthase dimerization. Mitochondria of wild-type cells lacking mtDNA contain a reduced number of cristae [43,46,67,99] and frequently contain inner membrane septa [102], resembling the ultrastructural phenotypes caused by absence of Mgm1. Thus, at least some inner membrane defects observed in Δ*mgm1* mutants could be a secondary consequence of loss of mtDNA. However, the analysis of temperature-sensitive mutants provided strong arguments for a direct role of Mgm1 in the establishment of mitochondrial architecture. Shortly after shift to non-permissive temperature mitochondria of the *mgm1–5* mutant show appearance of inner membrane septa [42,44] and loss of normal cristae [42]. Strikingly, this effect precedes the loss of mtDNA, and normal cristae structure is restored when the cells are returned to lower temperature [42]. This would be unexpected if loss of mtDNA was the primary cause of mitochondrial ultrastructure alterations. Furthermore, a reduction of cristae abundance has been observed *in vitro* in isolated organelles of *mgm1–7* and *mgm1–10* mutants [44]. Together, these results suggest that Mgm1 is directly involved in cristae formation.

Mgm1 is present in mitochondria in two different isoforms [157,158]. The long isoform (l-Mgm1) is an integral protein of the inner membrane with the major part facing the intermembrane space [170]. Proteolytic cleavage by the rhomboid protease Pcp1 generates the short isoform (s-Mgm1) [170,171], which lacks the transmembrane domain [170,172]. Thus, s-Mgm1 is a soluble intermembrane space protein that is peripherally associated with the membrane [170]. The generation of the two isoforms is coordinated during import of newly synthesized Mgm1 into mitochondria and tightly coupled to the matrix ATP level [172]. The situation is even more complex in human cells where the *OPA1* gene generates eight different mRNAs by alternative splicing [173]. All mRNA variants encode proteins with a cleavable mitochondrial targeting sequence. The resulting long forms (L-OPA1) contain an N-terminal transmembrane domain and are processed by the proteases YME1L and/or OMA1 to generate soluble short forms (S-OPA1) (reviewed in [174,175]).

Both isoforms of Mgm1 are required for normal mitochondrial network morphology in yeast, indicating that both are involved in inner membrane fusion [170]. GTPase activity of the short isoform is required for mitochondrial fusion *in vivo*, whereas GTPase activity of the long isoform appears to be dispensable [176,177]. It is currently unknown whether both Mgm1 isoforms are also required for normal mitochondrial ultrastructure. Electron microscopy of *pcp1* mutants, which contain low levels or no s-Mgm1, revealed ultrastructural defects which are somewhat similar to those reported for Δ*mgm1* strains [178]. This indicates that either both isoforms, or s-Mgm1 alone, could be required for maintenance of normal cristae structure in yeast. By contrast, L-OPA1 appears to be sufficient to maintain largely normal ultrastructure in MEFs [179–181]. Cristae defects were observed in *Phb2*^−/−^ MEFs, which lack L-OPA1 due to constitutive proteolytic processing into short forms, and expression of L-OPA1, but not S-OPA1, restored cristae structure [182]. This indicates that the long form could be required for normal cristae formation. However, expression of splice variants that generate only S-OPA1 appears to be sufficient to at least partially restore normal mitochondrial ultrastructure in *Opa1*^−/−^ MEFs [179,180]. Hence, more work is needed to elucidate the role of the long and short isoforms of Mgm1 and OPA1 in the formation of normal cristae structure.

The molecular mechanism of Mgm1 in the establishment and maintenance of cristae architecture is not completely understood. Mgm1 belongs to the family of dynamin-like proteins which comprises many large GTPases that are well known for their membrane remodelling capacities [183]. For OPA1 it has been proposed that hetero-oligomeric complexes composed of the short and long isoforms lace up crista junctions and counteract their opening during apoptosis [152,163]. Also in yeast, homo- and heterotypic interactions of the long and short isoforms of Mgm1 have been observed [176,177]. Strikingly, Mgm1 molecules residing in opposing membranes are capable of forming complexes in *trans*, suggesting that these interactions could aid the shaping of cristae by steadily linking adjacent membranes without fusing them [44,176]. Studies investigating Mgm1's localization by immunogold electron microscopy did not reveal an accumulation of Mgm1 at crista junctions. Instead, Mgm1 was found to be distributed along the entire inner membrane with a slight enrichment at the inner boundary membrane [9,42,177]. More specifically, s-Mgm1 preferentially localizes to the inner boundary membrane whereas the long isoform is more enriched in cristae [177]. The functional significance of this distribution is largely unknown. It has been suggested that in particular homotypic l-Mgm1 interactions in *trans* could play a role in inner membrane architecture by tethering adjacent cristae membranes [176].

The ability of s-Mgm1 to bind and remodel membranes could play a role in shaping the inner membrane. *In vitro* studies demonstrated that purified s-Mgm1 possesses lipid-binding activity [184] and assembles on the surface of liposomes into a homo-oligomeric lattice [176,185]. Assembly of s-Mgm1 causes liposome aggregation and tethering [185,186], the formation of s-Mgm1-decorated membrane tubes [186] and local membrane deformation which is stimulated by addition of GTP [187]. Recently, the crystal structures of the short Mgm1 isoforms from yeast and the filamentous fungus *C. thermophilum* were solved, revealing a lipid-binding domain that is connected to the G domain by a stalk [188,189]. *C. thermophilum* s-Mgm1 also assembles on membranes *in vitro* and causes liposome tubulation. Cryo-electron tomography revealed that it forms helical filamentous arrays on positively curved membranes [188]. Strikingly, *C. thermophilum* s-Mgm1 was found to assemble on membranes with either positive or negative curvature, indicating that it could bind to cristae membranes from the cristae lumen. A model was proposed that s-Mgm1 helices could form on cristae membranes and that a dynamin-like power stroke within these filaments could either constrict or widen the cristae, depending on the directionality of the helix (left- or right-handed) [188]. However, the observation that s-Mgm1 is enriched at the inner boundary membrane in yeast [177] does not support this exciting idea. Moreover, the *in vivo* situation is further complicated by s-Mgm1 not only forming homo- but also hetero-oligomeric complexes with l-Mgm1 [176,177]. Thus, it remains a challenge for the future to elucidate the mechanism by which Mgm1 helps to establish mitochondrial ultrastructure.

## 6. Mitochondrial phospholipids

It is well established that lipids influence important properties of the membrane, including thickness, packing density, net charge and curvature [190]. It is therefore tempting to speculate that they also contribute to the establishment of mitochondrial architecture. The main phospholipid classes of the yeast mitochondrial inner membrane are phosphatidylcholine (PC) and phosphatidylethanolamine (PE). In addition, a high amount of the mitochondrial signature lipid cardiolipin (CL) sets the inner membrane apart from other membranes of the cell. The sterol content (mostly ergosterol in yeast) is low [191,192]. In this section, we will review the evidence for a role of phospholipids in shaping the mitochondrial inner membrane in yeast. For a more detailed discussion of the importance of lipids, in particular cardiolipin, for various mitochondrial processes in several organisms the reader is referred to refs. [193–195].

**Figure 3.**
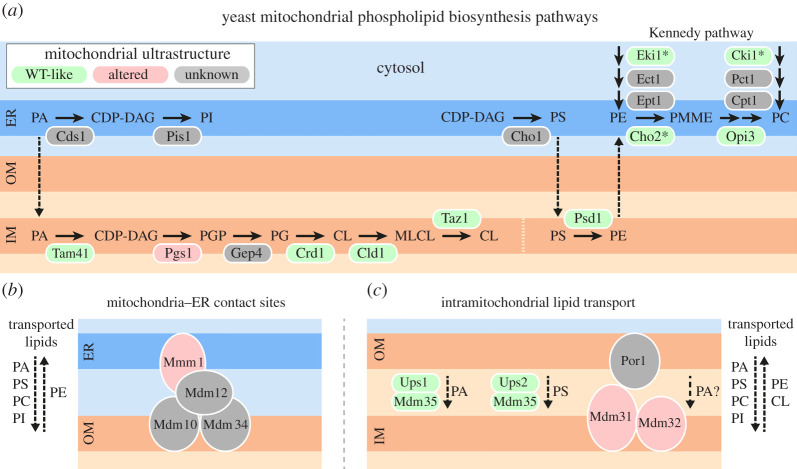
Mitochondrial lipid biosynthesis and transport pathways contribute to maintenance of mitochondrial ultrastructure. Cartoons represent (*a*) mitochondrial phospholipid biosynthesis pathways, and phospholipid exchange between (*b*) mitochondria and the ER or (*c*) both mitochondrial membranes. Dashed lines indicate lipid transport processes across different membranes. Please note that in (*a*) membrane association of the proteins is not illustrated. Different colours indicate whether absence of the functional protein has been reported to cause changes in mitochondrial ultrastructure, as indicated. In some cases, only information from double or triple mutants is available (highlighted with an asterisk). See text and table 2 for details. CDP-DAG, cytidine diphosphate-diacylglycerol; CL, cardiolipin; IM, mitochondrial inner membrane; MLCL, monolysocardiolipin; OM, mitochondrial outer membrane; PA, phosphatidic acid; PC, phosphatidylcholine; PE, phosphatidylethanolamine; PG, phosphatidylglycerol; PGP, phosphatidylglycerolphosphate; PI, phosphatidylinositol; PMME, phosphatidylmonomethylethanolamine; PS, phosphatidylserine; WT, wild-type.

Mitochondria harbour the CL biosynthetic pathway and are involved in the synthesis of PE (figure 3*a*) [195–197]. They have to import all other lipids from other organelles, mainly the ER, where most cellular lipids are synthesized. Conversely, mitochondria-derived PE is exported to the ER where it serves as a substrate for the formation of PC. Mitochondria are not part of the secretory pathway and depend on other routes of lipid distribution. The current model is that lipid transport mainly occurs at direct membrane contacts between mitochondria and other organelles, in particular the ER (figure 3*b*) [195,197]. The intramitochondrial distribution of lipids is mediated by intermembrane space-localized lipid transport proteins of the Ups/PRELI family, and presumably also direct contacts between the mitochondrial outer and inner membranes (figure 3*c*) [195,197].

In yeast, PC is highly abundant in most cellular membranes. It constitutes approximately 40% of all mitochondrial phospholipids, with a higher concentration that is connected to the outer membrane [191]. Surprisingly, yeast cells bearing mutations that affect PC biosynthesis are viable, even when cellular and mitochondrial PC levels are strongly reduced, and cristae structure is not notably affected in these strains [75,78]. This indicates that yeast cells can tolerate strong alterations of the mitochondrial lipid profile and preserve normal cristae structure under these conditions.

However, several yeast mutants are known to display both an altered mitochondrial lipid composition and altered mitochondrial ultrastructure (table 2 and figure 3). For example, mitochondria of mutants lacking Mmm1, a key component of the ERMES complex that physically connects mitochondria and the ER [138], have reduced PE and CL levels [63,68], increased ergosterol content [198], and an abnormal giant spherical morphology with strongly elongated cristae that may form loops and parallel stacks [67]. Similarly, cells lacking either of the two closely related inner membrane proteins Mdm31 and Mdm32 contain large swollen ball- or ring-like mitochondria [64,199], exhibit reduced mitochondrial PE and/or CL levels [63] and suffer from defects of mitochondrial ultrastructure with only few visible cristae and internal membranes forming circular structures within the matrix [64]. Mdm31 and Mdm32 have been suggested to play a role in Ups1-independent biosynthesis of CL [200], potentially by Mdm31 interacting with the outer membrane protein Por1 [201]. This might play a role in intramitochondrial transport of the precursor of CL biosynthesis, phosphatidic acid (PA) [201]. Furthermore, overexpression of *MDM33*, a gene encoding an inner membrane protein of unknown function, results in altered inner membrane structure [79,80] and decreased mitochondrial CL and PE levels [79]. Taken together, these observations suggest that lipids, in particular PE and CL, could play a role in maintenance of normal mitochondrial architecture in yeast.

CL and PE together make up approximately 40% of all phospholipids of the inner membrane. They are both cone-shaped because the hydrophilic headgroups are relatively small compared to the hydrophobic parts and belong to the group of non-bilayer lipids [194]. Cone-shaped lipids favour negative membrane curvature [190]. However, molecular dynamics simulations suggest that CL has a higher preference for curved membrane regions than PE [202]. These properties suggest that local accumulation of CL (and possibly PE) at negatively curved regions could stabilize or even induce high membrane curvature. This might be particularly important at the matrix-facing leaflet of crista junctions as well as the cristae lumen-facing leaflet of highly curved tubular cristae regions or tightly bent cristae rims [193]. In support of this model, elegant *in vitro* assays with giant liposomes demonstrated that these can form invaginations that resemble cristae upon local acid administration, but only when they contain CL [203]. Furthermore, CL accumulates at the curved poles and septal regions of bacterial cells [204,205] and highly curved membrane tubes generated *in vitro* from giant vesicles [206]. However, in mitochondria the formation of subdomains with different lipid composition at regions with particularly high membrane curvature remains to be demonstrated.

Yeast cells possess alternative pathways for the synthesis of PE (reviewed in [195,196,207]). They can synthesize it from diacylglycerol and ethanolamine via the Kennedy pathway in the cytosol and ER. Furthermore, they can generate it by decarboxylation of phosphatidylserine (PS). This is either mediated by Psd1 in the mitochondrial inner membrane or by Psd2, which is located in the endomembrane system. The precursor of mitochondrial PE synthesis, PS, is imported from the ER and transported between both mitochondrial membranes by Ups2 in complex with Mdm35 [71,208]. Of note, a fraction of Psd1 localizes not to mitochondria but to the ER [209] and it has been suggested that Psd1 localized in the mitochondrial inner membrane can also catalyse the conversion of PS to PE in the mitochondrial outer membrane at membrane contact sites [71].

In yeast, Psd1 activity appears to be the primary source for mitochondrial PE [210]. Accordingly, mitochondrial PE levels are reduced in mutants lacking Psd1 [63,76,77], Ups2 [63,65,66,69] or Mdm35 [63,65,66]. However, these mutants have no or only mild cristae defects. A slight decrease in cristae length has been noted for Δ*psd1* strains [75]. Similarly, Δ*mdm35* mutants show only mild defects of cristae formation [45]. The absence of Ups2 does not strongly affect mitochondrial ultrastructure [71], albeit the number of cristae is reduced [63]. Thus, although several lines of evidence point to an importance of non-bilayer lipids for mitochondrial membrane biology [194], a proof of a strong impact of PE on yeast mitochondrial ultrastructure is lacking so far.

The biosynthesis of CL takes place in the mitochondrial inner membrane (reviewed in [195,197,207]). It starts with PA, which is imported from the ER. Ups1 mediates the transport of PA between the outer and inner membrane. Like Ups2, also Ups1 acts in complex with Mdm35. In the inner membrane, PA is converted to phosphatidylglycerol (PG) in three consecutive reactions catalysed by Tam41, Pgs1 and Gep4 in yeast. The CL synthase Crd1 subsequently synthesizes CL from PG and cytidine diphosphate diacylglycerol (CDP-DAG).

Yeast cells lacking Ups1, Tam41, Pgs1, Gep4 or Crd1 show a strong reduction of mitochondrial CL levels [63,70]. However, only deletion of the *PGS1* gene is associated with severe mitochondrial ultrastructure defects. Δ*pgs1* mutants contain strongly elongated cristae, inner membrane septa and mitochondria with multiple layers of internal circular membranes. By contrast, cristae structure is inconspicuous in Δ*ups1* and Δ*tam41* mutants [70]. Δ*crd1* strains show increased cristae length but only few mitochondria display an aberrant ultrastructure [72]. It is currently unknown why Δ*pgs1* mutants suffer from altered cristae structure, while other CL biosynthesis mutants contain rather normal mitochondria. In sum, it appears that normal mitochondrial ultrastructure can be maintained even when CL levels are strongly reduced.

Acyl chains of newly synthesized CL are remodelled by the concerted action of the specific phospholipase Cld1, which removes acyl chains from CL, and the transacylase Taz1 (Tafazzin), which regenerates CL [195,197]. Together, these two enzymes thereby determine the final acyl chain composition of CL. Mitochondria from Δ*taz1* mutants contain less CL and more monolysocardiolipin, whereas Δ*cld1* mutants show normal mitochondrial CL levels but an altered CL acyl chain composition [72,73]. Initially, Δ*taz1* yeast mutants were reported to occasionally contain swollen mitochondria and elongated and aberrantly shaped cristae membranes [74]. However, a careful investigation of the Δ*cld1* and Δ*taz1* mutants revealed that cristae structure was mostly unaffected in both cases. Cristae length was found to be increased, although in both cases only in one of the two tested strain backgrounds, and a moderately increased number of mitochondria with altered ultrastructure were observed [72]. Overall, these results suggest that in yeast both CL and CL remodelling are not strictly required for the establishment of normal mitochondrial ultrastructure.

However, evidence from other organisms suggests that CL might contribute to cristae structure in metazoan mitochondria. Alterations of mitochondrial ultrastructure have been observed upon loss of Tafazzin function in mouse differentiated cardiomyocytes [211] and in the flight muscle from *Drosophila melanogaster* [211,212]. So why is this not apparent in yeast? The answer is most likely to lie in functional redundancy. Yeast cells can tolerate loss of either mitochondrial PE or CL synthesis, but simultaneous absence of both pathways is lethal [213]. This suggests that both phospholipids have overlapping functions and that loss of mitochondrial PE or CL can be compensated by the presence of the other lipid.

Besides the headgroup also the acyl chain composition impacts on the overall shape and properties of phospholipids. Thus, changes of membrane properties may not necessarily be deducible from the abundance of each phospholipid class without taking the acyl chain composition into account. It has been shown that PC depletion is accompanied by an increase in the relative amount of PE in yeast. At the same time, PE acyl chain composition changes with saturated and shorter acyl chains becoming more abundant. This presumably serves to counteract membrane property changes caused by increasing levels of the non-bilayer lipid PE [214]. Together, this indicates that functional redundancy of lipid classes and compensatory mechanisms, such as acyl chain remodelling, help yeast cells to cope with alterations of their membrane lipid composition. If this is sufficient to maintain the overall properties of the membrane, this probably explains the absence of strong ultrastructural phenotypes even when the lipid profiles of mitochondrial membranes are strongly altered.

Taken together, the shape of the mitochondrial inner membrane is very likely to depend on membrane lipid homeostasis. Consistently, several mutants that display altered mitochondrial lipid composition exhibit abnormal cristae structure (table 2). However, it is unknown whether the observed ultrastructural defects are direct consequences of altered lipid profiles. Surprisingly, several mutants with strongly altered lipid composition show no or only mild ultrastructural phenotypes (see figure 3 and table 2). This is probably due to functional redundancy of different lipid classes and compensatory mechanisms. Clearly more work is needed to elucidate the precise mechanisms of lipid homeostasis in the maintenance of mitochondrial membrane shape in yeast.

## 7. Functional interplay of the pathways

An intimate coordination of several activities is necessary to build the complex architecture of the mitochondrial inner membrane. ATP synthase dimers align along the edges of cristae membranes to establish and maintain their highly curved shape. MICOS is located at crista junctions, establishes contacts to the mitochondrial outer membrane, and has inner membrane-shaping activities. Dynamin-related GTPases, Mgm1/OPA1, mediate inner membrane fusion and remodel the shape of cristae membranes by an as yet poorly understood mechanism. The machinery mediating synthesis and trafficking of lipids adjusts the membrane lipid composition to fit the curvatures of cristae membranes. Numerous observations indicate that the activities of these pathways are highly interconnected and depend on each other.

The activities of ATP synthase dimerization and MICOS are interconnected in multiple ways. Mutants lacking factors required for ATP synthase dimerization and MICOS subunits show positive genetic interactions, such that deletion of one gene (partially) rescues the deletion phenotype of the other gene [38,215]. Furthermore, cells lacking Mic60 show increased levels of ATP synthase supercomplexes, whereas overexpression of Mic60 results in decreased levels of ATP synthase supercomplexes, indicating antagonistic activities [38]. Mutants simultaneously lacking ATP synthase dimers and one or more MICOS subunits are largely devoid of cristae and sometimes contain inner membrane septa [42,46,53]. Intriguingly, Mic10 selectively binds to ATP synthase dimers, suggesting a mechanism for the coordination of MICOS activity and ATP synthase oligomerization [129,216,217].

In many organisms, including yeast and humans, the mitochondrial genome encodes major subunits of the membrane-embedded F_o_ part of the ATP synthase. Therefore, all mutants lacking mtDNA are devoid of the cristae-shaping activity of ATP synthase dimerization. As Δ*mgm1* deletion mutants are unable to maintain mtDNA [155] they are lacking both Mgm1 and ATP synthase dimer dependent membrane remodelling activities. Loss of mtDNA in mitochondrial fusion mutants can be suppressed by deletion of the *DNM1* gene required for mitochondrial fission [218,219]. However, attempts to construct respiratory competent Δ*dnm1* Δ*mgm1* Δ*atp21* or Δ*dnm1* Δ*mgm1* Δ*mic60* Δ*atp21* mutants failed, suggesting that the simultaneous loss of Mgm1 and ATP synthase dimers is deleterious for mitochondrial function and/or maintenance of mtDNA [42].

Δ*dnm1* Δ*mgm1* and Δ*dnm1* Δ*mic60* double mutants are able to maintain mtDNA and form cristae, albeit with abnormal structure. By contrast, mitochondria of Δ*dnm1* Δ*mgm1* Δ*mic60* triple mutants show relatively few internal sheet-like membranes and many mitochondrial profiles appear empty, even under conditions that allow maintenance of mtDNA [42,45]. This indicates that the simultaneous loss of Mgm1 and MICOS is deleterious for cristae formation.

Cristae formation and mitochondrial inner membrane lipid homeostasis mutually affect each other. An elegant *in vitro* study demonstrated that CL is required for Mgm1 to associate with liposomes and assemble into higher-order oligomers [176]. Mdm35 is a small intermembrane space protein required for transport of PA from the outer to the inner membrane of mitochondria, where PA is incorporated into CL [70]. Interestingly, Δ*dnm1* Δ*mdm35* Δ*mgm1* mitochondria have drastically altered inner membrane structure and are frequently devoid of cristae, a phenotype that is not seen in Δ*dnm1* Δ*mdm35* Δ*fzo1* mitochondria. This suggests that CL and Mgm1 play an overlapping function in cristae formation [45].

Crd1 is the CL synthase in the mitochondrial inner membrane. Δ*crd1* Δ*mic60* cells show reduced Mic27 stability, suggesting that the association of Mic27 to the Mic10 subcomplex is CL dependent and CL supports the localization of MICOS at crista junctions [53]. Accordingly, oligomerization of Mic10 is compromised in mitochondria of Δ*crd1* cells [56]. Furthermore, it was suggested that close contacts between the mitochondrial outer and inner membranes established by MICOS are required to organize membrane lipid biosynthesis in mitochondria [71]. Thus, it appears that mitochondrial lipids, most notably CL, are required to support the assembly of the protein complexes shaping the inner membrane. At the same time, efficient phospholipid trafficking and synthesis within mitochondria depend on the appropriate organization of mitochondrial architecture.

**Figure 4.**
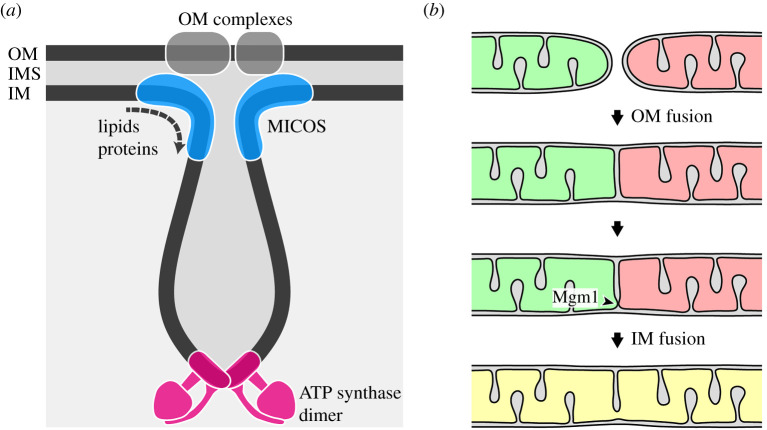
Two pathways contribute to mitochondrial cristae formation in yeast. (*a*) MICOS coordinates the establishment of crista junctions at contact sites with the outer membrane, and ATP synthase dimerization generates and/or maintains high membrane curvature at cristae rims and tubular cristae. (*b*) Inner membrane fusion by Mgm1 could be the initial step for formation of lamellar cristae. These are then shaped with the aid of MICOS and ATP synthase, similar to in (*a*). See text and ref. [42] for details. OM, mitochondrial outer membrane; IM, mitochondrial inner membrane; IMS, intermembrane space.

It is clear that the establishment and maintenance of cristae architecture is a highly complex process. Some models have been proposed to explain how MICOS, ATP synthase and Mgm1 cooperate to establish mitochondrial architecture. The antagonistic activities of Mic60 and ATP synthase dimerization suggest that MICOS is particularly important to establish negative membrane curvature at crista junctions, whereas ATP synthase dimers induce positive membrane curvature at cristae tips and rims. Thus, the ratio of MICOS and ATP synthase dimers is decisive in shaping cristae [38]. It is conceivable that the activities of these two pathways is sufficient for the formation of tubular cristae in yeast mitochondria [42] (figure 4*a*).

What might be the role of Mgm1? Mitochondria of Δ*dnm1* Δ*mgm1* double mutants maintain mtDNA and contain numerous cristae [42,43,45], demonstrating that cristae can be generated in the absence of Mgm1. Strikingly, electron tomography of temperature-sensitive *mgm1–5* mutant cells demonstrated that lamellar cristae are rapidly lost upon inactivation of Mgm1–5, whereas tubular cristae can be maintained. Consistently, Δ*dnm1* Δ*mgm1* mutant mitochondria contain exclusively tubular cristae pointing to a specific role of Mgm1 in the biogenesis of lamellar cristae [42]. Based on these observations it was proposed that Mgm1-dependent cristae formation occurs during mitochondrial fusion. Initially, fusion of the outer membranes might generate inner membrane septa. Mgm1 then initiates fusion of opposing inner membranes at these septa, and assembly of ATP synthase dimers shapes a lamellar inner membrane sac at this site. Inner membrane fusion is then halted at the boundary region by the assembly of MICOS complexes, thereby generating a crista junction [42] (figure 4*b*). Alternatively, inner membrane septa might be generated by inner membrane fission to initiate Mgm1-dependent formation of lamellar cristae in a similar way [45].

Taken together, the current literature suggests that there are at least two pathways of mitochondrial cristae formation. Formation of tubular cristae involves the assembly of crista junctions by MICOS and the generation of positive curvature by ATP synthase dimerization. Growth of tubular cristae depends on import of proteins and lipids into the inner membrane and their allocation to cristae, but is independent of Mgm1. Formation of lamellar cristae similarly involves MICOS for the assembly of crista junctions and ATP synthase dimers for the generation of membrane curvature at cristae rims. In addition, it requires Mgm1 to initiate cristae generation by fusion of inner membrane septa. While these models explain many observations reported in the literature, they have to be verified and refined by further experimentation. Also, it remains a challenge for the future to determine the contribution of mitochondrial lipid composition to mitochondrial cristae biogenesis, and to reveal the role of cristae in the distribution and inheritance of mtDNA. Clearly, the investigation of the pathways shaping the mitochondrial inner membrane will remain an exciting field of research in the coming years.

## Data accessibility

This article has no additional data.
